# Safety and efficacy of treating symptomatic, partial-thickness rotator cuff tears with fresh, uncultured, unmodified, autologous adipose-derived regenerative cells (UA-ADRCs) isolated at the point of care: a prospective, randomized, controlled first-in-human pilot study

**DOI:** 10.1186/s13018-020-01631-8

**Published:** 2020-03-30

**Authors:** Jason L. Hurd, Tiffany R. Facile, Jennifer Weiss, Matthew Hayes, Meredith Hayes, John P. Furia, Nicola Maffulli, Glenn E. Winnier, Christopher Alt, Christoph Schmitz, Eckhard U. Alt, Mark Lundeen

**Affiliations:** 1 Sanford Orthopedics & Sports Medicine Sioux Falls, 1210 W. 18th St., Suite G01, Sioux Falls, SD 57104 USA; 2 Sanford Health, Sioux Falls, SD USA; 3 Sanford Radiology Clinic, Sioux Falls, SD USA; 4 SUN Orthopedics of Evangelical Community Hospital, Lewisburg, PA USA; 5 Department of Musculoskeletal Disorders, Faculty of Medicine and Surgery, University of Salerno, Salerno, Italy; 6 Centre for Sports and Exercise Medicine, Barts and The London School of Medicine and Dentistry, Mile End Hospital, Queen Mary University of London, London, UK; 7 School of Pharmacy and Bioengineering, Guy Hilton Research Centre, Keele University School of Medicine, Stoke on Trent, UK; 8 InGeneron, Inc., Houston, TX USA; 9 InGeneron GmbH, Munich, Germany; 10 Institute of Anatomy, Faculty of Medicine, LMU Munich, Munich, Germany; 11 Isar Klinikum, Munich, Germany; 12 Sanford Orthopedics & Sports Medicine Fargo, Fargo, ND USA

**Keywords:** Adipose-derived regenerative cells (ADRCs), Partial rotator cuff tear, Point of care treatment, Safety, Shoulder disease, Stem cells, Stromal vascular fraction

## Abstract

**Background:**

This study tested the hypothesis that treatment of symptomatic, partial-thickness rotator cuff tears (sPTRCT) with fresh, uncultured, unmodified, autologous adipose-derived regenerative cells (UA-ADRCs) isolated from lipoaspirate at the point of care is safe and more effective than corticosteroid injection.

**Methods:**

Subjects aged between 30 and 75 years with sPTRCT who did not respond to physical therapy treatments for at least 6 weeks were randomly assigned to receive a single injection of an average 11.4 × 10^6^ UA-ADRCs (in 5 mL liquid; mean cell viability: 88%) (*n* = 11; modified intention-to-treat (mITT) population) or a single injection of 80 mg of methylprednisolone (40 mg/mL; 2 mL) plus 3 mL of 0.25% bupivacaine (*n* = 5; mITT population), respectively. Safety and efficacy were assessed using the American Shoulder and Elbow Surgeons Standardized Shoulder Assessment Form (ASES), RAND Short Form-36 Health Survey, and pain visual analogue scale (VAS) at baseline (BL) as well as 3 weeks (W3), W6, W9, W12, W24, W32, W40, and W52 post treatment. Fat-saturated T2-weighted magnetic resonance imaging of the shoulder was performed at BL as well as at W24 and W52 post treatment.

**Results:**

No severe adverse events related to the injection of UA-ADRCs were observed in the 12 months post treatment. The risks connected with treatment of sPTRCT with UA-ADRCs were not greater than those connected with treatment of sPTRCT with corticosteroid injection. However, one subject in the corticosteroid group developed a full rotator cuff tear during the course of this pilot study. Despite the small number of subjects in this pilot study, those in the UA-ADRCs group showed statistically significantly higher mean ASES total scores at W24 and W52 post treatment than those in the corticosteroid group (*p* < 0.05).

**Discussion:**

This pilot study suggests that the use of UA-ADRCs in subjects with sPTRCT is safe and leads to improved shoulder function without adverse effects. To verify the results of this initial safety and feasibility pilot study in a larger patient population, a randomized controlled trial on 246 patients suffering from sPTRCT is currently ongoing.

**Trial registration:**

Clinicaltrials.gov ID NCT02918136. Registered September 28, 2016, https://clinicaltrials.gov/ct2/show/NCT02918136.

**Level of evidence:**

Level I; prospective, randomized, controlled trial.

## Background

Partial-thickness rotator cuff tears (PTRCT) are a common cause of shoulder pain, loss of function, and occupational disability [1–3]. They are classified according to location (articular, bursal, interstitial), grade (grade 1, < 3 mm deep; grade 2, 3–6 mm deep; grade 3, > 6 mm deep), and tear area [4]. Cadaveric and magnetic resonance imaging (MRI) studies reported the incidence of PTRCT between 13 and 25%, with an increasing incidence with age [5–7] and a higher incidence of articular-sided PTRCT than bursal-sided PTRCT [1, 8]. The etiology and pathogenesis of PTRCT are multifactorial and comprise several intrinsic factors (including age-related hypocellularity and decreased tissue vascularity) as well as extrinsic factors (including subacromial impingement, glenohumeral instability, internal impingement, and trauma) [1–3]. The higher incidence of articular-sided PTRCT may be related to the fact that the articular side of the rotator cuff is less vascularized than the bursal side [1].

According to the Guideline on Optimizing the Management of Rotator Cuff Problems of the American Academy of Orthopedic Surgeons [9, 10], the strength of recommendation for or against many nonoperative treatment options for rotator cuff tears and rotator cuff-related symptoms (including activity modification, exercise programs, use of non-steroidal anti-inflammatory drugs, and corticosteroid injections) has remained “inconclusive,” with no differentiation between bursal-sided and articular-sided PTRCT. Subacromial injection of corticosteroid provides short-term pain relief, but may not modify the course of the disease [11]. Steroid injections, commonly used in clinical practice, are not without risks. Dexamethasone (which has a 25 times higher anti-inflammatory potency than hydrocortisone [12]) may induce non-tenocyte differentiation of human tendon stem cells, potentially leading to tendon rupture [13] (c.f. also [14]). In line with this, a recent systematic review showed that pre-operative corticosteroid injections are correlated with an increased risk of revision surgery after rotator cuff repair in a temporal and dose-dependent manner [15]. Most authors agree that surgical treatment of PTRCT is generally indicated in patients with failure of nonoperative management for 3–6 months [1, 3]. Surgery, while generally successful, has some drawbacks, including the potential for complications, a lengthy recovery, and some authors report that it may not be better than conservative management [16]. Arthroscopic repair of bursal-sided and articular-sided PTRCT showed similar functional outcome and the same retear rate (approximately 10% during the first two years after surgery) [17, 18].

Over the past decade, stem cell injection therapy has emerged as a promising treatment for many musculoskeletal conditions. In animal models, injections of adult stem cells isolated from adipose tissue into pathologic rotator cuff tissues has been shown to produce a number of beneficial effects, including decreased number of inflammatory cells, improved regeneration of tendons with less scarred healing, improved collagen fiber arrangement, higher load-to-failure, and higher tensile strength of the treated tendons [19–23]. However, corresponding clinical studies have not yet been reported. Stem cells must not be confused with platelet-rich plasma (PRP). The latter is a component of blood that contains high levels of platelets which release growth factors for tissue repair [24]. Two recent reports (a meta-analysis and a double-blinded, randomized controlled trial (RCT)) concluded that injection of PRP is not beneficial in non-operative treatment of rotator cuff disease [25, 26].

Recent studies demonstrate the advantages of newer proprietary methods for harvesting and isolating stem cells [27–30]. This pilot study evaluated the safety and efficacy of treating symptomatic, bursal-sided, and articular-sided PTRCT (sPTRCT) which did not respond to physical therapy treatments for at least 6 weeks with a single injection of fresh, uncultured, unmodified, autologous adipose-derived regenerative cells (UA-ADRCs) isolated at the point of care (i.e., at the same location where harvesting of adipose tissue and injection of UA-ADRCs were carried out). The hypotheses were that (i) treatment of sPTRCT with UA-ADRCs does not result in any serious adverse event in the 12 months post treatment (primary clinical outcome), and (ii) compared to subjects who received a single subacromial injection of corticosteroid, subjects who received injection of UA-ADRCs show better function of the shoulder and greater reduction in pain at 24 and 52 weeks post treatment (secondary clinical outcome).

## Methods

### Study design

**Fig. 1:**
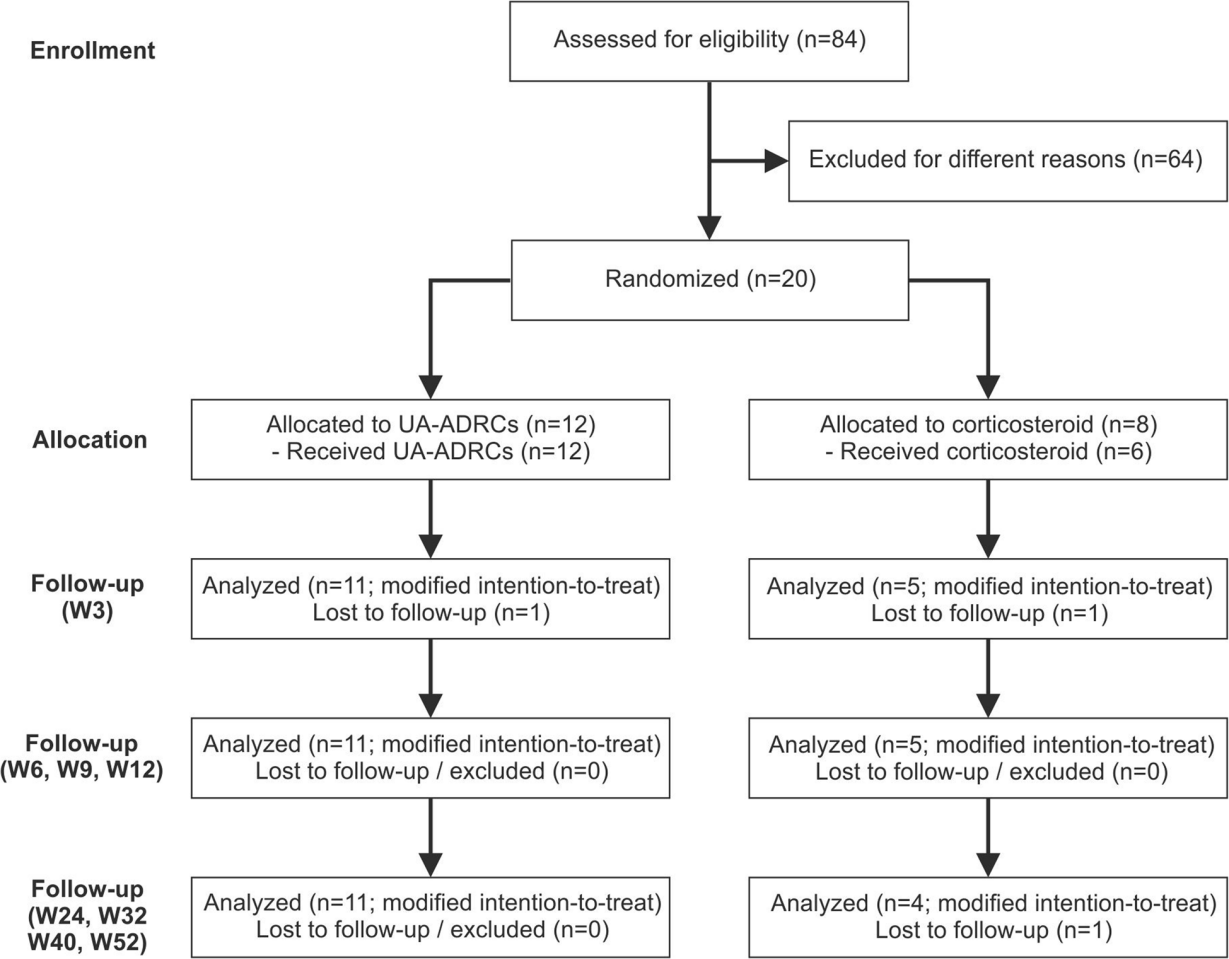
Flow of subjects in this pilot study according to CONSORT [31]

This was a first *in vivo*, two center, prospective, open-label, randomized controlled pilot study comparing UA-ADRCs injection and corticosteroid injection for the management of sPTRCT not responsive to at least 6 weeks of physical therapy treatments. All subjects were recruited from Sanford Orthopedics & Sports Medicine Sioux Falls (Sioux Falls, SD, USA) and Sanford Orthopedics & Sports Medicine Fargo (Fargo, ND, USA) between December 2016 and May 2017. Figure 1 shows the flow of subjects through this pilot study according to the CONSORT statement [31], and Table 1 the schedule of enrollment, interventions, and assessments according to SPIRIT [32]. Because this was a first-in-human pilot study, it was designed as an open-label trial, with treatment up to 30 days after screening and randomization. Subjects were allowed to withdraw their informed consent to participate in this pilot study at any time.

**Table 1:** Schedule of enrollment, interventions, and assessments during this pilot study according to SPIRIT [32]

Study period						
	Enrollment/Allocation	Post-allocation	Follow-up			Close-out
Timepoint	D0	D0–D30	W3^a^, W6^a^, W9^a^, W12^a^	W24^a^	W32^a^, W40^a^	W52^a^
Enrollment						
Clinical evaluation	X					
Eligibility screen	X					
Allocation	X					
MRI scan		X^b^		X		X
Interventions						
Injection of UA-ADRCs		X				
Injection of corticosteroid		X				
Assessments						
A, G, BH, BW, O, L	X					
Safety		X	X	X	X	X
ASES	X		X	X	X	X
RAND Short Form-36	X		X	X	X	X
Pain	X		X	X	X	X

*D* day; *W* week; *UA*-*ADRCs* fresh, uncultured, unmodified, autologous adipose derived regenerative cells; *A* age; *G* gender; *BH* body height; *BW* body weight; *O* occupation; *L* leisure activities

^a^Week after treatment

^b^The baseline MRI scan of one subject was performed 36 days before screening

### Ethics

This pilot study has received approval from the Institutional Review Board of Sanford Health (Sanford IRB #3 registration number 00007985) in accordance with the Declaration of Helsinki, and Investigational Device Exemption from the U.S. Food and Drug Administration (FDA) (no. 16956). This pilot study was registered on September 28, 2016 at Clinicaltrials.gov, with ID NCT02918136.

### Participants

Female and male adults aged 30–75 years with a 3T MRI diagnosis formulated by two musculoskeletal radiologists (M.H., M.H.) of PTRCT plus diagnosis of sPTRCT by an orthopedic shoulder specialist (J.L.H., M.L.) based on history and clinical examination (including but not limited to shoulder pain, shoulder muscle weakness, and limited shoulder range of motion) who had not responded to physical therapy treatments for at least 6 weeks were eligible for inclusion. All subjects had MRI scans that demonstrated PTRCT > 50% in the supraspinatus tendon (articular-sided, bursal-sided, or interstitial partial thickness tear, respectively). The MRI scans were reviewed by the musculoskeletal radiologists and the treating orthopedic shoulder specialist, and had to correlate with findings from clinical examination to come to the diagnosis of sPTRCT. For example, subjects with MRI diagnosis of PTRCT but pain mostly over their biceps groove and positive provocative tests for biceps pain (Yergason’s, Speed’s, Obrien’s tests [33]) were diagnosed with biceps pathology rather than sPTRCT (in this case, the PTRCT was not the primary symptom generator). Furthermore, subjects with PTRCT and pain primarily arising from subacromial impingement who, based on the experience of the musculoskeletal radiologists and the orthopedic shoulder specialists, could be assumed that injection of UA-ADRCs would not help were excluded from this pilot study. These decisions were always made on an individual basis rather than the presence of a certain stage of shoulder impingement [34]. The full list of inclusion and exclusion criteria is shown in Table 2. The vast majority of subjects assessed for eligibility to be enrolled in this pilot study were representative for citizens of U.S. Midwestern metropolitan areas. Subjects in both groups were recruited from the same population over the same period of time.

**Table 2:** Inclusion and exclusion criteria of subjects with symptomatic, partial-thickness rotator cuff tear enrolled in this pilot study

Inclusion criteria	
Males and females 30–75 years of age.	
Clinical symptoms consistent with a rotator cuff lesion including but not limited to pain, muscle weakness, or active-limited range of motion.	
Subjects who have not responded to physical therapy treatments for at least six weeks.	
Subjects with > 70% passive range of motion.	
Diagnosed with > 50% tear to supraspinatus muscle or < 5 mm separation assessed by MRI.	
Diagnosed with a partial-thickness rotator cuff tear.	
The ability of subjects to give appropriate consent.	
Exclusion criteria	
Age < 30 or > 75.	
Diagnosed with a full-thickness rotator cuff tear.	
Insufficient amount of subcutaneous tissue to allow recovery of 50 mL of lipoaspirate.	
History of systemic malignant neoplasms within last 5 years.	
History of local neoplasm within the last 6 months and any history of local neoplasm at site of administration.	
Subject is receiving immunosuppressant therapy or has known immunosuppressive or severe autoimmune disease that requires chronic immunosuppressive therapy (e.g., human immunodeficiency virus, systemic lupus erythematosus, etc.).	
Subjects who are known to be human immunodeficiency virus positive.	
Patients who have received a corticosteroid injection in rotator cuff site within last 3 months.	
Severe arthrosis of the glenohumeral or acromioclavicular joint.	
Irreparable rotator cuff tear (including rotator cuff tear arthropathy).	
Fatty atrophy above grade 2 in affected shoulder.	
Previous shoulder surgeries in affected shoulder.	
Any contraindication to MRI scan according to MRI guidelines, or unwillingness to undergo MRI procedures.	
History of tobacco use within the last 3 months.	
Patient is on an active regimen of chemotherapy.	
Patients with a documented history of liver disease or an alanine aminotransferase value >400.	
Allergy to sodium citrate of any “caine” type of local anesthetic.	
Patient is pregnant or breast feeding.	
Subject is, in the opinion of the investigator or designee, unable to comply with the requirements of this pilot study protocol or is unsuitable for this pilot study for any reason. This includes completion of Patient Reported Outcome instruments.	
Subject is currently participating in another clinical trial that has not yet completed its primary endpoint.	
Subject is part of a vulnerable population who, in the judgment of the investigator, is unable to give Informed Consent for reasons of incapacity, immaturity, adverse personal circumstances or lack of autonomy. This may include: individuals with mental disability, persons in nursing homes, children, impoverished persons, persons in emergency situations, homeless persons, nomads, refugees, and those incapable of giving informed consent. Vulnerable populations also may include members of a group with a hierarchical structure such as university students, subordinate hospital and laboratory personnel, employees of the sponsor, members of the armed forces, and persons kept in detention.	
Uncooperative patients or those with neurological/psychiatric disorders who are incapable of following directions or who are predictably unwilling to return for follow-up examinations.	

### Randomization and blinding

A total of 84 subjects suffering from sPTRCT were assessed. Before randomization, 64 of 84 subjects assessed for eligibility chose to withdraw, declined to sign consent, or were excluded because they did not meet the inclusion criteria or met any of the exclusion criteria. The remaining 20 subjects were randomly assigned to receive a single injection of UA-ADRCs (*n* = 12) or a single injection of methylprednisolone (*n* = 8). Randomization was performed using a computerized random-number generator to formulate subject allocation. The 20 subjects were randomized into six blocks. The person who determined whether a subject was eligible for inclusion in this pilot study was unaware, when this decision was made, of which group the subject would be allocated to. One subject in the UA-ADRCs group was treated but excluded from this pilot study immediately after treatment because the cell product failed to meet release criteria. Furthermore, two subjects in the corticosteroid group withdrew consent after randomization but prior to treatment, and another subject in this group withdrew consent shortly after treatment. Accordingly, the modified intention-to-treat (mITT) population comprised *n* = 11 subjects in the UA-ADRCs group and *n* = 5 subjects in the corticosteroid group (Fig. 1).

One subject in the corticosteroid group developed a full-thickness rotator cuff tear (considered a treatment-related serious adverse event) after the examination that took place 12 weeks after treatment and was lost to follow-up. This resulted in full analysis of 11/11 (100%) of the subjects in the UA-ADRCs group and 4/5 (80%) of the subjects in the corticosteroid group (mITT population) (Fig. 1). Missing data of the subject who was lost to follow-up were handled using the last observation carried forward method [35].

Characteristics of subjects in the mITT population at baseline are displayed in Table 3.

**Table 3:** Characteristics of included subjects at baseline (modified intention-to-treat population)

Variable	UA-ADRCs group (*n* = 11)	Corticosteroid group (*n* = 5)
Age, years, median; mean (SD; min; max)	64.6; 62.3 (9.6; 40; 74)	57.6; 57.3 (6.2; 47; 63)
Woman, *n* (%)	3 (27.3)	0 (0)
Body weight, kg, median; mean (SD; min; max)	93.9; 88.6 (18.1; 51.6; 111.1)	106; 104.1 (24.8; 74.5; 133.7)
Body height, cm, median; mean (SD; min; max)	178; 176 (8.8; 157; 188)	178; 178 (4.8; 173; 185)^a^
Affected shoulder, right (%)	9 (81.8)	3 (60.0)
ASES total score, median; mean (SD; min; max)	57; 58.7 (19.2; 30; 92)	50; 50.6 (15.0; 30; 65)
RAND Short Form-36 total score, median; mean (SD; min; max)	604; 557 (134; 270; 695)	560; 523 (90; 425; 627)
VAS pain score, median; mean (SD; min; max)	1.9; 2.6 (2.5; 0; 7.1)	4.1; 4.1 (2.5; 0.8; 7.4)
Tear volume, mm^3^, median; mean (SD; min; max)	47.3; 58.6 (37.4; 19.8; 128.9)	31.7; 28.7 (10.8; 14.6; 43.2)
Partial-thickness tear in supraspinatus tendon, *n* (%)	11 (100)	5 (100)
Location of tear, articular; bursal; interstitial (*n*)		
Occupation and leisure activities	Hairstylist (sewing), construction/dry wall (volleyball, watersports, hunting), retired (golf, walking, hunting), retired (shoveling, mowing, yard work), retired (remodeling homes), customer service/phone (walking, biking), customer service/computer work (none), pharmacist (golf, paddleboarding, water sports), truck driver (bowling), attorney (flying), farmer (family)	Driver (golf), ultrasound technician (golf, hunting, fishing), commercial real estate (golf, biking), office/patrol (basketball, hunting, fishing), pilot (cycling, jogging, light weights)

^a^Body height was not taken from one subject in the corticosteroid group

The subjects, physicians who performed the treatment, and assessors who performed baseline and follow-up examinations were not blinded in this pilot study. On the other hand, the physicians who analyzed MRI scans were blinded.

### Interventions

Subjects in the UA-ADRCs group had an outpatient syringe liposuction procedure performed by a licensed physician using a modified Coleman method [36]. To this end, either the periumbilical abdominal area, bilateral flanks, or medial thigh, respectively, were surgically disinfected. Then, local anesthesia was achieved by infiltrating the subcutaneous adipose tissue with an average of 316 ± 25 mL (mean ± standard error of the mean; SEM; range, 175–400) of modified Klein tumescent solution [37] (1051 mL of modified Klein tumescent solution consisted of 1000 mL lactated Ringer’s solution (E8000; Braun Medical, Irvine, CA, USA), 50 mL of 1% lidocaine (Hospira, Lake Forest, IL, USA), and 1 mL of 1:1000 epinephrine (Hospira)). Twenty minutes later, a stab incision was produced, and lipoaspiration was performed using a four-hole blunt tipped cannula (3 mm × 150 mm) (Shippert Medical Technologies, Centennial, CO, USA) and a 60 cm^3^ Luer-Lock syringe (VAC160, Merit Medical, South Jordan, UT, USA). After liposuction, manual pressure was applied to the wounds. Then, the wounds were closed using adhesive bandage strips (Curity or Dermacea Abdominal Pad; Covedian, Mansfield, MA, USA).

**Fig. 2:**
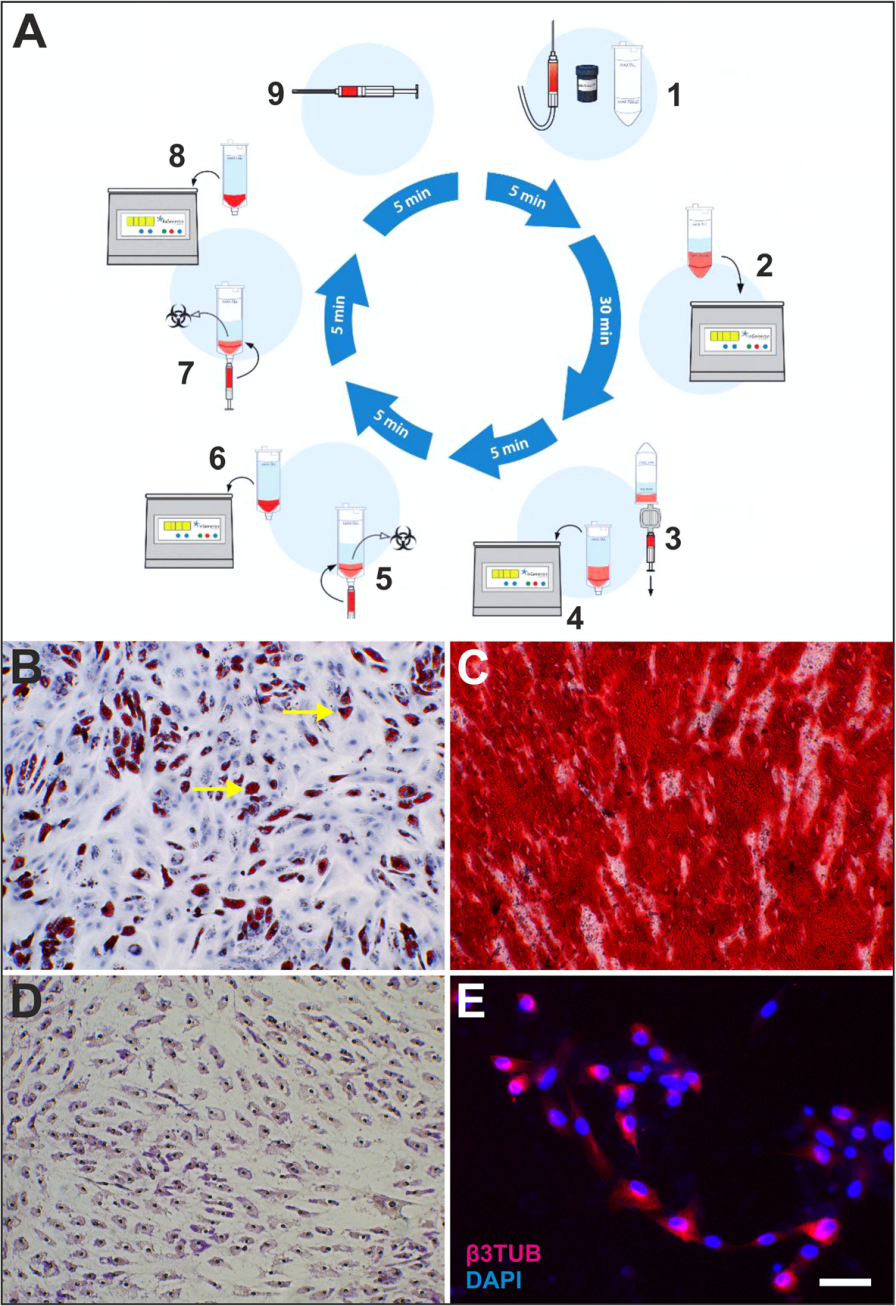
**a** Schematic representation of isolating UA-ADRCs from lipoaspirate with the Transpose RT/Matrase system (InGeneron) used in this pilot study (derived from [28]): (1) recovered lipoaspirate (25 mL) is loaded together with 2.5 mL reconstituted, proprietary enzymatic Matrase Reagent and lactated Ringer solution (preheated to 39 °C) into a processing tube up to the MAX FILL line; (2) the filled processing tubes are subjected in an inverted position inside the Transpose RT system to repetitive acceleration and deceleration for 30 min at 39 °C; (3) the processed lipoaspirate solution is filtered through a 200 μm filter and transferred into a wash tube; (4) after filling the wash tube with saline (room temperature) up to the MAX FILL line, the cells are separated from the rest of the tissue by centrifugation at 600 g for 5 min at room temperature; (5) the ADRCs (approximately 2 mL) are extracted through a swabable luer vial adapter at the bottom of the wash tube, and the remaining substances (fat, debris, and liquid) are discarded; (6) the cells are returned into the empty wash tube and (after adding fresh saline up to the MAX FILL line) centrifugated again for 5 min; (7, 8) the previous washing step is repeated; and (9) finally the concentrated ADRCs (approximately 3 mL) are extracted and slowly pushed through a luer coupler into a new sterile syringe for further application to the subject. This gentle and highly efficient isolation process results in a high cell yield (7.2 × 10^5^ ± 0.90 × 10^5^ ADRCs per mL lipoaspirate in [28]), high cell viability (85.9 ± 1.1% in [28]) and, thus, high number of living cells per mL lipoaspirate (6.25 × 10^5^ ± 0.79 × 10^5^ ADRCs per mL lipoaspirate in [28]). To our knowledge, the latter is the highest value ever reported in studies describing methods for isolating ADRCs [28]. **b**–**e** Adipogenic (**b**), osteogenic (**c**), hepatogenic (**d**), and neurogenic (**e**) differentiation potential of adipose derived stem cells (ASCs) derived from ADRCs isolated from lipoaspirate using the Transpose RT/Matrase system, demonstrated by culturing ASCs on their 3rd (**b**–**d**) or 6th passage (**e**) for 2 weeks (**b**, **c**), 10 days (**d**), or 3 weeks (**e**) in adipogenic (**b**), osteogenic (**c**), hepatogenic (**d**), or neurogenic (**e**) differentiation medium (panels taken from [28]). Adipogenic, osteogenic, hepatogenic, and neurogenic differentiation was demonstrated by respectively presence of intracytoplasmic lipids (triglycerides) using Oil red-O staining (**b**; the yellow arrows in **b** indicate single Oil red-O positive cells), presence of calcific deposits using Alizarin red staining (**c**), presence of structures containing a high proportion of carbohydrate macromolecules (glycogen, glycoprotein, and proteoglycans) using Periodic Acid Schiff staining (**d**), or immunofluorescence for the detection of beta III Tubulin (β3TUB) (**e**) (details are provided in [28]). The scale bar in E represents 100 μm in (**b**, **c**) and 50 μm in (**d**, **e**)

The harvested lipoaspirate (50 mL per subject) was processed with the Transpose RT / Matrase system (InGeneron, Houston, TX, USA) [28–30] to isolate UA-ADRCs (Fig. 2). The lipoaspirate was divided into two aliquots of 25 mL each. Then, each aliquot was incubated together with Matrase Reagent (InGeneron) for 30 min. The latter was performed in the Transpose RT processing unit under agitation at 37 °C according to the manufacturer’s instructions. The total procedure time was 70 min. The average cell yield (i.e., the number of cells isolated per gram of tissue) was 2.3 ± 0.2 × 10^5^ cells/g, and the average cell viability was 88 ± 3% (all data are related to the mITT population). Details of the final cell suspension are provided in Table 4.

**Table 4:** Characteristics of UA-ADRCs applied in this pilot study (modified intention-to-treat population)

Variable	Values	Final product acceptance criterion according to protocol
Cell number, × 10^6^; median; mean (SD; min; max)	10.4; 11.4 (3.1; 8.6; 20)	≥ 5×10^6^
Cell yield^a^, × 10^5^/g; median; mean (SD; min; max)	2.1; 2.3 (0.6; 1.7; 4.0)	≥ 1.0×10^5^/g
Cell viability, %; median; mean (SD; min; max)	92.3; 88.5 (8.7; 75; 97.3)	> 70%
Endotoxin, Equivalent Unit (EU) total^b^, median; mean (SD; min; max)	1.0; 1.0 (0.8; 0; 2.23)^**c**^	< 2.5 EU total
Gram stain, negative (%)	11 (100)	Negative

^a^Number of cells isolated per gram of tissue processed by the Transpose RT/Matrase system (InGeneron)

^b^In the final cell suspension

^c^These values were calculated without the data of one subject whose final cell suspension contained 60.5 EU total. In this case, the amount of endotoxin was miscalculated at release. This was discovered after the subject had already completed the follow-up examinations. The incident was reported to FDA who accepted the Corrective and Preventive Action and the report

No more than 2 h after the lipoaspirate was harvested, each subject in the UA-ADRCs group received a single injection that averaged 11.4 × 10^6^ UA-ADRCs in 5 mL liquid (mITT population). Subjects in the corticosteroid group received a single injection of 80 mg of methylprednisolone (40 mg/mL; 2 mL; Hospira) plus 3 mL of 0.25% bupivacaine (Hospira). Corticosteroid injections were made into the subacromial space and injections of UA-ADRCs were made into the tendon defect. All injections were made by a qualified physician under ultrasound guidance.

Subjects were instructed to avoid strenuous shoulder exertion and overhead activity for the first 24–48 h after treatment, continue with any home exercise program the therapists had already given them before enrollment, and increase their activities as tolerated. Specific restrictions were not placed.

### Outcome measurements and assessments

The primary clinical outcome was the occurrence of adverse events, defined as any untoward or unfavorable medical occurrence in a subject, including any abnormal sign, symptom, or disease, temporally associated with the subject’s participation in this pilot study, whether or not considered related to the subject’s participation in this pilot study. Possible adverse events, the occurrence of which was specifically considered, included (i) complications of liposuction and the injection procedure itself (the most important possible local adverse events that could occur on the skin and soft tissue at the puncture/injection site were pain, nerve damage, bruising, bleeding, infection, redness, swelling, tenderness, lightening, and thinning; the most important possible adverse events that could occur at the shoulder joint were shoulder pain, worsening of existing shoulder pain, joint infection, and tendon weakening; and the most important possible general symptoms were fever and inflammatory flare), and (ii) complications of injection of UA-ADRCs or corticosteroid (the most important treatment-related adverse events were tendon weakening and progression of sPTRCT into symptomatic full-thickness rotator cuff tear). Occurrence of adverse events was assessed immediately after treatment (i.e., injection of UA-ADRCs or corticosteroid) and 3 weeks (W3), W6, W9, W12, W24, W32, W40, and W52 post treatment. In addition, subjects were instructed to immediately report any potential adverse event regardless of the time of occurrence.

Secondary clinical outcomes were changes in the (i) American Shoulder and Elbow Surgeons (ASES) Society standardized shoulder assessment form (ASES total score), (ii) RAND Short Form-36 total score (c.f [38, 39].), (iii) Visual Analogue Scale for pain (VAS pain score), and (iv) size of the PTRCT measured on MRI scans as a function of time compared to baseline.

The ASES assessment form shows strong correlation with multiple rotator cuff specific scores, and has excellent reliability, construct validity, and responsiveness [40, 41]. The RAND Short Form-36 is a global measure of health-related quality of life that measures eight scales: physical functioning, role physical, bodily pain, general health, vitality, social functioning, role emotional, and mental health [42].

Pain was assessed in two ways. First, the ASES pain score was scaled from 10 (pain as bad as it can be) to 0 (no pain at all). The specific question asked was about the “Intensity of Pain” in the shoulder with no time reference given to guide the subject. Accordingly, the question suggested the overall severity of pain when experienced. Second, a validated VAS was used. The VAS is a very specific term that classically refers to an unidimensional line (i.e., an unmarked line that is 100 mm in length) to measure intensity of pain. The ends are defined as the extreme limits of the parameter to be measured orientated from the left (worst) to the right (best). This scale was embedded in the RAND Short Form-36 in this pilot study. The specific question asked was “What is your level of pain in your shoulder today?”

MRI evaluation of PTRCT was performed in the coronal, sagittal, and axial plane with T2 proton density-weighted, fat-saturated (PD FS) sequences (repetition time (TR) between 2375 and 3917 ms; echo time (TE) between 34 and 72 ms; NEX between 1.0 and 2.0; slice thickness between 3.0 and 4.0 mm, image size 320 × 320 or 512 × 512, respectively). MRI scans were performed using a MAGNETOM Skyra 3T (Siemens Medical Solutions USA, Malvern, PA, USA) or Signa Architect 3.0 T (GE Healthcare, Waukesha, WI, USA), respectively. The size of the PTRCT was measured in all three directions, and the tear volume was calculated as an ellipsoid from these data [43].

The ASES total score, RAND Short Form-36 total score, and VAS pain score were assessed at baseline (BL) and W3, W6, W9, W12, W24, W32, W40, and W52 post treatment; MRI scans were performed at BL, W24, and W52 post treatment.

### Power analysis

Because this was a first-in-human pilot study with occurrence of adverse events as primary clinical outcome, a small sample size was selected that was not based on a power analysis. Rather, the ultimate goal of this pilot study was to collect safety data that are sufficient to develop an appropriate pivotal study after 6 months of data collection for all subjects. This pivotal study, which finally will include 246 subjects with sPTRCT, is now recruiting (Clinicaltrials.gov ID NCT03752827 [44]).

### Statistical analysis

Mean and standard error of the mean (SEM) of all investigated variables (ASES total score, RAND Short Form-36 total score, VAS pain score, and size of PTRCT) were calculated. Differences between the groups were tested using two-way repeated measures ANOVA, with subject-specific values obtained at BL, W24, and W52 post treatment as matched data (values obtained at W3, W6, W9, W12, W32, and W40 post treatment were not considered in the statistical analysis). Post hoc Bonferroni tests were used for pairwise comparisons. In all analyses, an effect was considered statistically significant if its associated *p* value was smaller than 0.05. Calculations were performed using GraphPad Prism (Version 8.0.0 for Windows; GraphPad Software, San Diego, CA, USA).

## Results

### Safety of treating symptomatic, partial-thickness rotator cuff tear with UA-ADRCs

Table 5 provides a detailed overview on all treatment emergent adverse events (TEAEs) that were reported during the course of this pilot study (coded with the Medical Dictionary for Regulatory Activities (MedDRA) Version 19.1 [45]). The total number of TEAEs was 34 in the UA-ADRCs group and 13 in the corticosteroid group. All subjects in both groups reported experiencing at least one TEAE. The number of subjects with 1/2/3/4/5/6/7 TEAEs was 1/3/4/2/0/0/1 in the UA-ADRCs group (3.1 ± 0.5; median, 3) and 2/0/2/0/1/0/0 in the corticosteroid group (2.6 0.8; median, 3). These data were not significantly different between the groups (Mann-Whitney test; *p* = 0.622).

**Table 5:** Number of treatment emergent adverse events (TEAEs) and number of subjects with TEAE by system organ class and preferred term (coded with the Medical Dictionary for Regulatory Activities (MedDRA) Version 19.1 [45])

Adverse event category	Days post treatment	Group	Severity	Probability
Cardiac disorders				
Coronary artery disease	21	Cg	Mild	Not related
Myocardial infarction	91	Ug	Severe	Not related
Myocardial infarction	126	Ug	Severe	Not related
Gastrointestinal disorders				
Abdominal discomfort	86	Ug	Mild	Not related
Abdominal pain	0	Ug	Mild	Possible
Abdominal pain	0	Ug	Mild	Possible
Dental necrosis	35	Ug	Moderate	Unlikely
Dysphagia	242	Ug	Moderate	Not related
General disorders and administration site conditions				
Chest pain	149	Cg	Mild	Not related
Infections and infestations				
Bronchitis	231	Ug	Moderate	Not related
Diverticulitis	299	Ug	Moderate	Not related
Pharyngitis	4	Cg	Mild	Unlikely
Pharyngitis	133	Ug	Mild	Not related
Sinusitis	49	Ug	Mild	Not related
Staphylococcal infection	141	Ug	Mild	Not related
Viral upper respiratory tract infection	14	Ug	Mild	Possible
Injury, poisoning and procedural complications				
Concussion	40	Ug	Moderate	Not related
Contusion	29	Ug	Mild	Not related
Ligament sprain	35	Cg	Mild	Not related
Tendon rupture	30	Cg	Moderate	Not related
Tooth fracture	164	Cg	Moderate	Not related
Musculoskeletal and connective tissue disorders				
Arthralgia (knee)	8	Ug	Mild	Not related
Arthralgia (hip)	114	Cg	Mild	Not related
Back pain	33	Ug	Mild	Not related
Musculoskeletal pain	22	Ug	Mild	Unlikely
Musculoskeletal pain	43	Cg	Moderate	Possible
Musculoskeletal pain	44	Ug	Mild	Unlikely
Musculoskeletal pain	49	Cg	Moderate	Possible
Musculoskeletal pain	64	Cg	Mild	Unlikely
Musculoskeletal pain	158	Cg	Mild	Unlikely
Musculoskeletal pain	168	Ug	Mild	Not related
Musculoskeletal pain	224	Ug	Severe	Not related
Pain in extremity	3	Ug	Moderate	Possible
Pain in extremity	120	Ug	Mild	Not related
Pain in extremity	208	Ug	Mild	Not related
Progression of sPTRCT into sFTRCT	114	Cg	Severe	Definite
Tendonitis	58	Ug	Moderate	Not related
Neoplasms benign, malignant and unspecified (incl cysts and polyps)				
Seborrhoeic keratosis	126	Ug	Mild	Not related
Psychiatric disorders				
Alcoholism	139	Ug	Mild	Not related
Renal and urinary disorders				
Dysuria	19	Ug	Mild	Not related
Respiratory, thoracic and mediastinal disorders				
Chronic obstructive pulmonary disease	296	Ug	Moderate	Not related
Cough	147	Cg	Mild	Not related
Dyspnea	27	Ug	Moderate	Unlikely
Dyspnea	364	Ug	Moderate	Not related
Rhinitis allergic	65	Ug	Mild	Not related
Skin and subcutaneous tissue disorders				
Nail discoloration	126	Ug	Mild	Not related
ascular disorders				
Essential hypertension	129	Ug	Mild	Not related

*Cg* corticosteroid group, *Ug* UA-ADRCs group, *sPTRCT* symptomatic partial-thickness rotator cuff tear, *sFTRCT* symptomatic full-thickness rotator cuff tear

Four different subjects in the UA-ADRCs group (one subject each on day 22 [D22], D44, D168, and D224 post treatment) and four different subjects in the corticosteroid group (one subject each at D43, D49, D64, and D158 post treatment) reported musculoskeletal pain. Furthermore, in the UA-ADRCs group, two different subjects reported pain in extremity (one subject at D208 post treatment, and another subject at D3 and again at D120 post treatment, with different extremities affected at D3 and D120), another two different subjects reported abdominal pain (both immediately post treatment), and another two different subjects reported dyspnea (one subject each on D27 and D364 post treatment).

The number of TEAEs classified as *mild*/*moderate*/*severe* was 21/10/3 in the UA-ADRCs group and 8/4/1 in the corticosteroid group. The three severe TEAEs in the UA-ADRCs group were myocardial infarction (one subject on D91 and again at D126 post treatment) and musculoskeletal pain (another subject at D224 post treatment). None of the severe TEAEs were related to treatment. The severe TEAE in the UA-ADRCs group was treatment-related progression of sPTRCT into symptomatic full-thickness rotator cuff tear.

The relationship of TEAEs to treatment classified as *Not related*/*Unlikely*/*Possible*/*Probable*/*Definite* was 26/4/4/0/0 in the UA-ADRCs group and 7/3/2/0/1 in the corticosteroid group. The *definite* TEAE (one subject in the corticosteroid group) was progression of sPTRCT into symptomatic full-thickness rotator cuff tear. Those TEAEs that were classified as *possible* in the UA-ADRCs group were mild abdominal pain (two subjects immediately post treatment; most probably because of the liposuction procedure), moderate pain in extremity (another subject on D3 post treatment), and mild viral upper respiratory tract infection (another subject on D14 post treatment), and in the corticosteroid group moderate musculoskeletal pain (two subjects on D43 and D49 post treatment).

Except for one subject, the amount of endotoxin in the final cell suspension of all subjects in the UA-ADRCs group was below 2.5 equivalent units (EU) total (Table 4), which was the final product acceptance criterion according to protocol. The final cell suspension of one subject contained a total of 60.5 EU of endotoxin. In this subject, the amount of endotoxin was miscalculated at release; this was discovered after the subject had already completed the follow-up examinations. The incident was reported to the FDA who accepted the Corrective and Preventive Action and the report. The following TEAEs were reported for this subject: musculoskeletal pain on day 22 (D22) post treatment (mild; relationship to treatment classified as *unlikely*) and myocardial infarction on D91 and D126 post treatment (severe; *not related*).

Gram stain of the final cell suspension of all subjects in the UA-ADRCs group showed a negative result prior to cell administration. Furthermore, 14-day cultures of ten out of the 11 final cell suspensions of these subjects displayed a negative finding. The 14-day culture of the final cell suspension of one subject evidenced the presence of *Propionibacterium acnes*. The TEAEs reported for this subject were viral upper respiratory tract infection on D14 post treatment (mild; *possible*), dyspnea on D27 post treatment (moderate; *unlikely*), allergic rhinitis on D65 post treatment (mild; *not related*), and essential hypertension on D129 post treatment (mild; *not related*).

### Efficacy of treating symptomatic, partial-thickness rotator cuff tear with UA-ADRCs

**Fig. 3:**
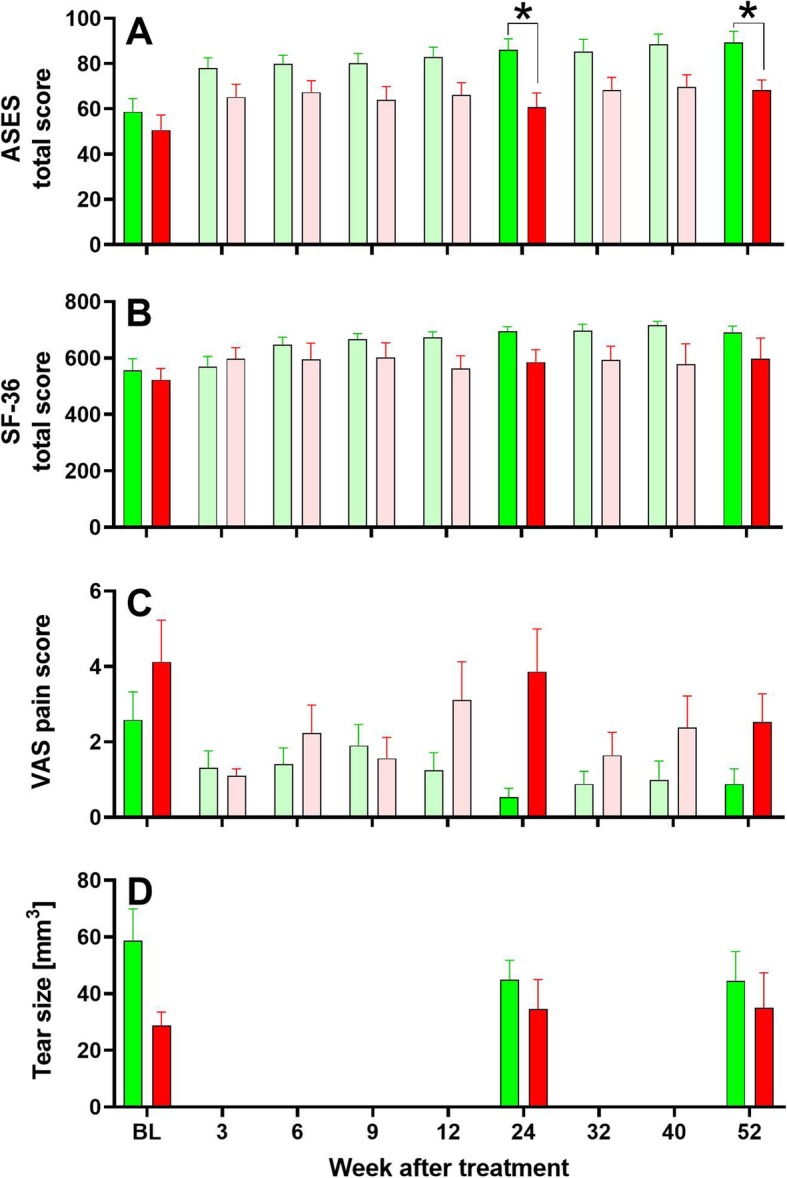
Mean and standard error of the mean of ASES total score (**a**), RAND Short Form-36 total score (**b**), VAS pain score (**c**), and tear size (**d**) as a function of time post treatment after treating sPTRCT with a single injection of UA-ADRCs isolated from lipoaspirate at the point of care (green bars) or a single injection of corticosteroid (red bars). Results of statistical analysis (post-hoc Bonferroni's multiple comparisons test) are indicated (*p < 0.05). Note: bars with pale color show those data that were not considered in the statistical analysis (according to the hypothesis tested in this pilot study)

With regard to efficacy of treating sPTRCT with respectively a single injection of UA-ADRCs isolated from adipose tissue at the point of care or a single injection of corticosteroid (secondary clinical outcome of this pilot study), Fig. 3 shows mean and SEM of ASES total score, RAND Short Form-36 total score, VAS pain score, and size of the PTCRT as a function of time post treatment. Results of the statistical analysis (*p* values) of these data are summarized in Table 6.

**Table 6:** Results of the statistical analysis of the efficacy data

Analysis/Variable	ASES total score	RAND Short Form-36 total score	VAS pain score	Tear size
Two-way RM ANOVA				
p_Time_	**< 0.001**	**0.006**	**0.040**	0.811
p_Treatment_	**0.017**	0.088	0.011	0.237
p_Time × Treatment_	0.199	0.425	0.249	0.305
P_Subject_	**0.008**	**0.005**	**0.024**	**< 0.001**
Post-hoc Bonferroni’s multiple comparisons test				
p_BL_	> 0.009	> 0.009	0.847	0.090
p_W24_	**0.033**	0.195	0.124	> 0.999
p_W52_	**0.022**	0.835	0.306	> 0.999

*P* values < 0.05 are given boldface

The mean ASES total score of the subjects in the UA-ADRCs group increased from 58.7 ± 5.8 (mean ± SEM) at BL to 86.1 ± 4.9 at W24 and 89.4 ± 4.9 at W52 post treatment, and of the subjects in the corticosteroid group from 50.6 ± 6.7 at BL to 60.8 ± 6.2 at W24 and 68.4 ± 4.4 at W52 post treatment (Fig. 3a). Differences between the groups at W24 and W52 were statistically significant (*p* < 0.05).

The mean RAND Short Form-36 total score of the subjects in the UA-ADRCs group increased from 557 ± 40.1 at BL to 696 ± 15.7 at W24 and 691 ± 22.6 at W52 post treatment, and of the subjects in the corticosteroid group from 523 ± 40.4 at BL to 586 ± 44.0 at W24 and 599 ± 72.2 at W52 post treatment (Fig. 3b). Differences between the groups were not statistically significant (*p* > 0.05).

The mean VAS pain score of the subjects in the UA-ADRCs group decreased from 2.6 ± 0.7 at BL to 0.5 ± 0.2 at W24 and 0.9 ± 0.4 at W52 post treatment, and of the subjects in the corticosteroid group from 4.1 ± 1.1 at BL to 3.9 ± 1.1 at W24 and 2.5 ± 0.8 at W52 post treatment (Fig. 3c). As in case of the mean RAND Short Form-36, the total score differences between the groups were not statistically significant (*p* > 0.05).

The mean tear size of the subjects in the UA-ADRCs group decreased from 58.6 ± 11.3 mm^3^ at BL to 45.0 ± 6.8 mm^3^ at W24 and 44.5 ± 10.3 mm^3^ at W52 post treatment, and of the subjects in the corticosteroid group increased from 28.7 ± 4.8 mm^3^ at BL to 34.6 ± 10.4 at W24 and 35.0 ± 12.4 at W52 post treatment (Fig. 3d). Again, the differences between the groups were not statistically significant (*p* > 0.05).

## Discussion

To our knowledge, this is the first report of sPTRCT treated with UA-ADRCs. We evidenced the following key findings: (i) no severe adverse events related to the injection of UA-ADRCs in the 12 months after treatment; (ii) no greater risks than those connected with treatment of sPTRCT with corticosteroid injection; and (iii) subjects in the UA-ADRCs group showed statistically significantly higher mean ASES total scores at W24 and W52 post treatment than subjects in the corticosteroid group (*p* < 0.05).

### Safety of treating symptomatic, partial-thickness rotator cuff tear with UA-ADRCs

The safety profile of treating sPTRCT with UA-ADRCs presented here, which was the primary clinical outcome of this pilot study, was based on the following three pillars: (i) application of UA-ADRCs rather than other types of stem cells; (ii) enzymatic rather than non-enzymatic isolation of UA-ADRCs; and (iii) use of the Transpose RT/Matrase system (InGeneron [28–30];) rather than other systems for enzymatic isolation of UA-ADRCs. With respect to the second and third pillar, Aronowitz and colleagues [46] proposed to judge a system or method for isolating UA-ADRCs by the following factors: nucleated cell count, nucleated cells per milliliter of tissue processed, cellular viability, level of residual enzymatic activity, data from flow cytometry and CFU-F assay, infection control, ease of use, cost to operate, and processing time. Except for cost to operate, all these aspects were addressed for the Transpose RT/Matrase system in detail in a recent experimental study [28] (c.f. Fig. 2).

### Efficacy of treating symptomatic, partial-thickness rotator cuff tear with UA-ADRCs

**Fig. 4:**
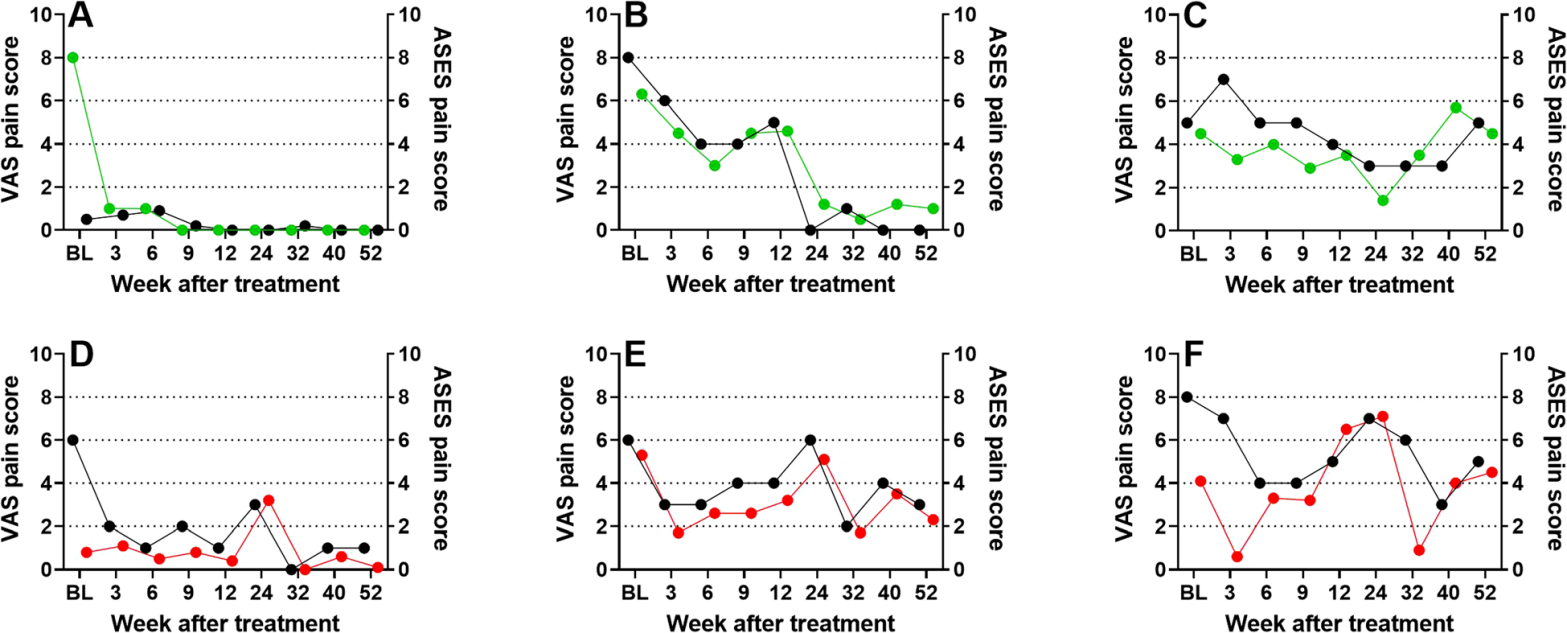
Individual ASES pain scores (black dots and right *Y* axis) and VAS pain scores (green and red dots and left *Y* axis) of select subjects treated with respectively UA-ADRCs (**a–c**) or corticosteroid (**d**–**f**) in this pilot study. Details are provided in the main text.

Demonstration of the efficacy of treating sPTRCT with UA-ADRCs was only the secondary clinical outcome of this pilot study, which justified the small sample of subjects and the limited selection of clinical examination methods. Next to the finding of higher mean ASES total scores at W24 and W52 after injection of UA-ADRCs than after injection of corticosteroid, the data of this pilot study reinforce the need to exactly describe which questions were asked about subjects’ pain (ASES pain score in this pilot study: “Intensity of Pain” in the shoulder with no time reference given to guide the subject; VAS in this pilot study: “What is your level of pain in your shoulder today?”). In this context, Fig. 4 shows comparisons of the individual time course of ASES pain score and VAS pain score of select subjects in this pilot study. Specifically, Figure 4a–c represents one subject each treated with UA-ADRCs and respectively substantial deviation between the two pain scores at baseline but fast recovery after treatment (Fig. 4a), almost identical pain scores and slower recovery after treatment (Fig. 4b), or dissociation between the two pain scores but no recovery over time (Fig. 4c). Figure 4d–f represents one subject each treated with corticosteroid and similar time course. These data do not only justify to present separate VAS pain scores (next to ASES pain scores as part of ASES total scores) in this pilot study, but emphasize the need to precisely describe the questions that were asked when assessing subjects’ pain in studies with pain as relevant endpoint.

**Fig. 5:**
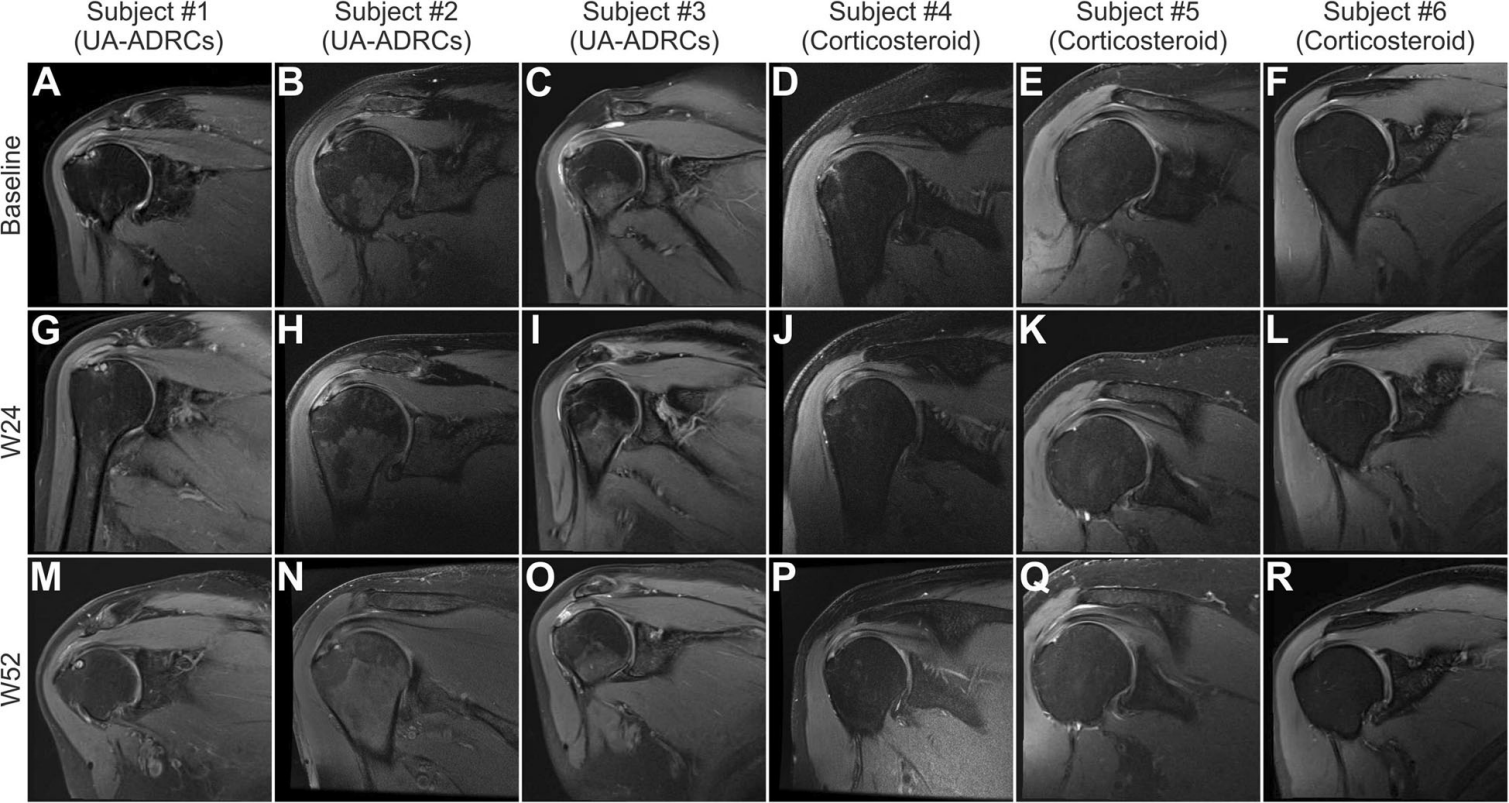
Coronal MRI scans (T2, proton density-weighted, fat-saturated (PD FS)) of the index shoulder of select subjects treated with respectively UA-ADRCs (**a**–**c**, **g**–**i**, **m**–**o**) or corticosteroid (**d**–**f**, **j**–**l**, **p**–**r**) in this pilot study. Repetition time (TR) was 2375 ms in **n**, 2950 ms in **d**, 3317 in **j**, 3500 in **a**, **c**, **e**, **f**, **i**, **k**, **m**, **o**, **q**, **r**, 3516 in **p**, 3660 in **l**, 3820 in **g**, and 3917 in **b**, **h**. Echo time (TE) was 34 ms in **a**, **c**, **e**, **f**, **g**, **i**, **k**–**m**, **o**, **q**, **r**, 37 in **n**, 45 in **b**, **h, j**, 46 in **d,** and 65 in **p**. NEX was 1 in **a**–**m**, **o**, **q**, **r**, 1.5 in **p,** and 2 in **m**

The results of the MRI analysis performed in this pilot study (Fig. 3d) may suggest that treatment of sPTRCT with UA-ADRCs did not result in complete healing up to 1 year post treatment. In this context, Fig. 5 shows MRI scans of select subjects in this pilot study. Specifically, Fig. 5 represents one subject each treated with UA-ADRCs whose PTRCT showed respectively complete healing (Fig. 5a, g, m), partial healing (Fig. 5b, h, n), or initial healing followed by worsening over time (Fig. 5c, i, o), as well as one subject each treated with corticosteroid whose PTRCT showed respectively almost complete healing (Fig. 5d, j, p), worsening over time (Fig. 5e, k, q), or only a very small tear (Fig. 5f, l, r). However, a more differentiated consideration appears necessary in this regard. Most importantly, the question of complete healing would have required to analyze biopsies of the treated tendons, which was not covered by the protocol of this pilot study. A recent case report [47] evaluating the outcome of treatment of sPTRCT with UA-ADRCs indicated that MRI scans do not necessarily reflect tendon regeneration evidenced by histologic evaluation of a biopsy taken from the treated tendon.

With regard to the potential mechanisms of action of UA-ADRCs in the treatment of sPTRCT, it is crucial to bear in mind that, in contrast to adipose derived stem cells (ASCs), UA-ADRCs in principle cannot be labeled. Accordingly, it is not possible to experimentally (or even clinically) determine whether the following benefits of ASCs also apply to UA-ADRCs, although it is reasonable to hypothesize that this is indeed the case. Specifically, ASCs can stay locally, survive and engraft in the new host tissue into which the cells were applied [48], differentiate under guidance of the new microenvironment into cells of all three germ layers [28] (c.f. Fig. 2), integrate into and communicate within the new host tissue by forming direct cell-cell contacts [27], exchange genetic and epigenetic information through release of exosomes [27], participate in building new vascular structures in the host tissue [27, 29, 30], positively influence the new host tissue by release of cytokines (among them vascular endothelial growth factor and insulin-like growth factor 1) [49], protect cells at risk in the new host tissue from undergoing apoptosis [49], and induce immune-modulatory and anti-inflammatory properties [50, 51]. In any case, the combination of these mechanisms of action apparently render UA-ADRCs more powerful in the treatment of sPTRCTs than corticosteroid.

Treatment of subjects suffering from sPTRCT with stem cells has been reported in several studies [52–56]. However, for a variety of reasons (no control group, no randomization, augmentation of rotator cuff repair with stem cells, arthroscopic evaluation of the rotator cuff before injection of stem cells, use of bone marrow-derived mesenchymal stem cells (BM-MSCs), use of ASCs rather than UA-ADRCs), most of these studies and their outcome cannot be compared with this pilot study (summarized in Table 7). In particular, in four of these studies [52–55], stem cells were used to augment arthroscopic rotator cuff repair rather than as the only therapy (aside from physical therapy after initial treatment). An exception is a study by Jo et al. [56], in which subjects suffering from sPTRCT were treated with injection of 1 × 10^7^ ASCs (group 1; *n* = 3), 5 × 10^7^ ASCs (group 2; *n* = 3), or 1 × 10^8^ ASCs (groups 3 (*n* = 3) and 4 (*n* = 10)), respectively. Liposuction was performed 3 weeks before injection. During a follow-up period of 6 months after treatment, subjects in all groups showed a reduction in the mean Shoulder Pain and Disability Index (group 1: from 43 to 18; group 2: from 64 to 12; group 3: from 75 to 16) and VAS pain score (group 1: from 78 to 36; group 2: from 90 to 28; group 3: from 90 to 26) (all data are approximate values taken from graphical representation of data in [56]). Furthermore, subjects in groups 2 and 3 showed an improvement in the constant score during the follow-up period (groups 2: from 62 to 76; group 3: from 56 to 66). Statistical analysis did not include between groups comparison. Of note, all subjects in groups 1 and 2 suffered from bursal-sided PTRCT, whereas subjects in group 3 suffered from articular-sided PTRCT, and no group showed complete healing as evaluated with MRI scans and arthroscopic inspection at the end of the follow-up period. In summary, the results by Jo et al. [56] are in line with the results of this pilot study. However, they do not establish any advantage of ASCs over UA-ADRCs in the treatment of sPTRCT. Rather, one has to consider all the potential disadvantages of ASCs outlined above, which may also explain that the best results were obtained by Jo et al. [56] when injecting a number of ASCs that was a magnitude higher than the number of UA-ADRCs that was applied in this pilot study. Furthermore, a recent publication by the Lancet Commission on Stem Cells and Regenerative Medicine [57] highlighted that, when performing long-term expansion in culture, even under optimal conditions, cells are exposed to different kinds of stress (mechanic, oxidative) that could affect their safety as medicinal product (quoted from [57]).

**Table 7:** Comparison of key characteristics of this pilot study with other studies on treatment of subjects suffering from sPTRCT with stem cells published in the literature

Reference characteristic	This study	[52]	[53]	[54]	[55]	[56]
Use of stem cells as the sole therapy	Yes	No	No	No	No	Yes
Number of subjects in the treatment group	11	14	45	8	35	13
Control group	Yes	No	No	No	Yes	No^a^
Number of subjects in the control group	5	n/a	n/a	n/a	35	3+3
RCT	Yes	No	No	No	No	No
Statistical analysis of differences between and/or within groups as result of treatment	Yes (b/w)	No	Yes (w)	No	Yes (b/w)	Yes (w)
Augmentation of rotator cuff repair with stem cells	No	Yes	Yes	Yes	Yes	No
Arthroscopic evaluation of the rotator cuff before injection of stem cells	No	n/a	n/a	n/a	n/a	Yes
Use of UA-ADRCs (1), ASCs (2) or BM-MSCs (3)	1	3	3	3	1	2

*RCT* randomized controlled trial; *UA*-*ADRCs* fresh, uncultured, unmodified, autologous adipose derived regenerative cells; *ASCs* adipose-derived stem cells; *BM*-*MSCs* bone marrow-derived mesenchymal stem cells

^a^Different groups of subjects with different doses of ASCs, but no other control group

Some authors proposed bone marrow stimulation by drilling of holes into the proximal humerus at the footprint of arthroscopic rotator cuff repair [58–60]. However, the real benefit of these procedures in which mesenchymal stem cells (MSCs) may serve to augment arthroscopic rotator cuff repair rather than the only therapy has yet to be demonstrated (c.f [23].). The latter also applies to isolation of MSCs from the subacromial bursa, rotator cuff tendon, biceps tendon, or synovial fluid, respectively, as proposed by some authors (reviewed in [23]). To our knowledge, application of such MSCs in the management of PTRCT has not yet been reported. In any case, it appears not possible to isolate MSCs from the subacromial bursa, rotator cuff tendon, biceps tendon, and synovial fluid at the point of care.

Many studies tested treatment of rotator cuff injuries and sPTRCT with PRP (see [25, 26, 61–67] for the most recent systematic reviews, meta-analyses, and editorial comments in this regard). Earlier systematic reviews and meta-analyses showed no (or almost no) benefit of augmenting arthroscopic rotator cuff repair with PRP [61–64], but two very recent meta-analyses demonstrated that the use of PRP in rotator cuff repair resulted in improved functional outcomes, pain levels, and healing rates compared to control [65, 66]. In contrast, another very recent meta-analysis concluded that PRP injections may not be beneficial in nonoperative treatment of rotator cuff disease [25]. This is supported by a very recent, well-designed, double-blinded RCT on 80 adults with symptomatic isolated interstitial tears of the supraspinatus tendon which reported no improvement in clinical scores and tendon healing between injection of PRP or saline within the interstitial supraspinatus tears [26]. Even worse, injection of PRP was associated with more adverse events than injection of saline in the latter study [26]. One reason for the lack of benefit of PRP in nonoperative treatment of rotator cuff tears may be hypocellularity as an important intrinsic factor of the etiology and pathogenesis of PTRCT [1–3]. Considering the potential mismatch between growth factors released by PRP [24, 68] and an insufficient number of stem cells in PTRCT [1–3] to be stimulated by these growth factors, both from a conceptual point of view and based on the data discussed here application of UA-ADRCs appears to be the better option for treating sPTRCT.

### Limitations

Because this prospective, randomized, controlled pilot study on UA-ADRCs vs. corticosteroid for treating sPTRCT was a first-in-human pilot study with safety as primary clinical outcome, it was designed as open-label pilot trial: thus, it has a number of inherent limitations. Specifically, only a small sample of subjects suffering from sPTRCT was investigated, only a limited number of clinical examination methods was applied, no power analysis was carried out, and neither the subjects nor the physicians who performed treatment and the assessors who performed baseline and follow-up examinations were blinded (only the physicians who analyzed the MRI scans were blinded). Another limitation was that only one control treatment was evaluated. Alternatives could have been placebo injection or suprascapular nerve block. The latter was recently shown to result in better clinical outcome than subacromial injection of the same amount of 9 mL of 1% ropivacaine and 1 mL of betamethasone at W6 and W12 post treatment (follow-up beyond W12 was not reported) [69]. However, it was not the ultimate aim of this pilot study to conclusively establish UA-ADRCs as treatment of sPTRCT. Rather, the ultimate aim of this study was to collect safety data that are sufficient to develop an appropriate pivotal study which finally will include 246 subjects with sPTRCT. This pivotal study, which is not affected by the limitations outlined above, is now recruiting (Clinicaltrials.gov ID NCT03752827 [44]) on the basis of the very encouraging pilot data presented in this report.

## Conclusions

The results of this pilot study suggest that the use of UA-ADRCs in subjects with sPTRCT is safe and leads to improved shoulder function without adverse effects. Larger trials are necessary to verify this. Clinicians should consider UA-ADRCs instead of injection of corticosteroids or PRP. In the long run, treatment of sPTRCT with injection of UA-ADRCs may delay or even prevent surgical treatment of sPTRCT.

## Data Availability

The datasets used and analyzed during this pilot study are available from the corresponding author on reasonable request, taking into account any confidentiality.

## Abbreviations

A: Age
ASCs: Adipose-derived stem cells
ASES: American Shoulder and Elbow Surgeons
BH: Body height
BL: Baseline
BM-MSCs: Bone marrow-derived mesenchymal stem cells
BW: Body weight
C: Celcius
CG: Corticosteroid group
cm: Centimeters
cm^3^: Cubic centimeter
CONSORT: Consolidated Standards of Reporting Trials
D: Day
EU: Equivalent unit
FDA: Food and Drug Administration
FS: Fat saturated
g: Gram
G: Gender
IFATS: International Federation for Adipose Therapeutics and Science
kg: Kilograms
L: Leisure activities
max: Maximum
mg: Milligrams
min: Minimum
mL: Milliliters
mm^3^: Cubic millimeters
MRI: Magnetic resonance imaging
MSCs: Mesenchymal stem cells
mTT: Modified intention-to-treat
n: Number
NEX: Number of excitations
O: Occupation
PD: Proton density weighted
PRP: Platelet-rich plasma
PTRCT: Partial-thickness rotator cuff tear
RAND: Research and development
RCT: Randomized controlled trial
SD: Standard deviation
SEM: Standard error of the mean
SPIRIT: Standard protocol items: recommendations for interventional trials
sFTRCT: Symptomatic, full-thickness rotator cuff tear
sPTRCT: Symptomatic, partial-thickness rotator cuff tear
T: Tesla
TEAE: Treatment emergent adverse events
TE: Echo time
TR: Repetition time
UG: UA-ADRCs group
U.S.: United States of America
UA-ADRCs: Fresh, uncultured, unmodified, autologous adipose derived regenerative cells
VAS: Visual analogue scale
W: Week
%: Percent

## References

[1] Matava MJ, Purcell DB, Rudzki JR (2005). Partial-thickness rotator cuff tears. Am J Sports Med..

[2] Via AG, De Cupis M, Spoliti M, Oliva F (2013). Clinical and biological aspects of rotator cuff tears. Muscles Ligaments Tendons J.

[3] Matthewson G, Beach CJ, Nelson AA, Woodmass JM, Ono Y, Boorman RS, Lo IK, Thornton GM (2015). Partial thickness rotator cuff tears: current concepts. Adv Orthop..

[4] Ellman H (1990). Diagnosis and treatment of incomplete rotator cuff tears. Clin Orthop Relat Res..

[5] Cotton RE, Rideout DF (1964). Tears of the humeral rotator cuff. J Bone Joint Surg.

[6] Fukuda H (2000). Partial-thickness rotator cuff tears: a modern view on Codman's classic. J Shoulder Elbow Surg..

[7] Sher JS, Uribe JW, Posada A, Murphy BJ, Zlatkin MB (1995). Abnormal findings on magnetic resonance images of asymptomatic shoulders. J Bone Joint Surg Am..

[8] Payne LZ, Altchek DW, Craig EV, Warren RF (1997). Arthroscopic treatment of partial rotator cuff tears in young athletes: a preliminary report. Am J Sports Med..

[9] https://www.aaos.org/research/guidelines/rcp_summary.pdf [cited 10 February 2020].

[10] Checketts JX, Scott J, Gordon J, Jones J, Horn J, Farabough M, Whitener J, Boose M, Vassar M (2018). An evaluation of the rotator cuff repair research pipeline. Orthop J Sports Med..

[11] Coombes BK, Bisset L, Vicenzino B (2010). Efficacy and safety of corticosteroid injections and other injections for management of tendinopathy: a systematic review of randomised controlled trials. Lancet..

[12] https://clincalc.com/Corticosteroids/ [cited 10 February 2020].

[13] Zhang J, Keenan C, Wang JH (2013). The effects of dexamethasone on human patellar tendon stem cells: implications for dexamethasone treatment of tendon injury. J Orthop Res..

[14] Ramírez J, Pomés I, Cabrera S, Pomés J, Sanmartí R, Cañete JD (2014). Incidence of full-thickness rotator cuff tear after subacromial corticosteroid injection: a 12-week prospective study. Mod Rheumatol..

[15] Puzzitiello RN, Patel BH, Nwachukwu BU, Allen AA, Forsythe B, Salzler MJ. Adverse impact of corticosteroid injection on rotator cuff tendon health and repair: a systematic review. Arthroscopy. 2019. pii: S0749-8063(19)31198-3 [Epub ahead of print]. doi: 10.1016/j.arthro.2019.12.006. 10.1016/j.arthro.2019.12.006 31862292

[16] Kukkonen J, Joukainen A, Lehtinen J, Mattila KT, Tuominen EK, Kauko T, Aärimaa V (2014). Treatment of non-traumatic rotator cuff tears: a randomised controlled trial with one-year clinical results. Bone Joint J..

[17] Kim SJ, Kim SH, Lim SH, Chun YM (2013). Use of magnetic resonance arthrography to compare clinical features and structural integrity after arthroscopic repair of bursal versus articular side partial-thickness rotator cuff tears. Am J Sports Med..

[18] Kim KC, Shin HD, Cha SM, Park JY (2014). Repair integrity and functional outcome after arthroscopic conversion to a full-thickness rotator cuff tear: articular- versus bursal-side partial tears. Am J Sports Med..

[19] Kim SH, Chung SW, Oh JH (2014). Expression of insulin-like growth factor type 1 receptor and myosin heavy chain in rabbit's rotator cuff muscle after injection of adipose-derived stem cell. Knee Surg Sports Traumatol Arthrosc..

[20] Valencia Mora M, Antuña Antuña S, García Arranz M, Carrascal MT, Barco R (2014). Application of adipose tissue-derived stem cells in a rat rotator cuff repair model. Injury..

[21] Chen HS, Su YT, Chan TM, Su YJ, Syu WS, Harn HJ, Lin SZ, Chiu SC (2015). Human adipose-derived stem cells accelerate the restoration of tensile strength of tendon and alleviate the progression of rotator cuff injury in a rat model. Cell Transplant..

[22] Gumucio JP, Flood MD, Roche SM, Sugg KB, Momoh AO, Kosnik PE, Bedi A, Mendias CL (2016). Stromal vascular stem cell treatment decreases muscle fibrosis following chronic rotator cuff tear. Int Orthop..

[23] Tsekes D, Konstantopoulos G, Khan WS, Rossouw D, Elvey M, Singh J (2019). Use of stem cells and growth factors in rotator cuff tendon repair. Eur J Orthop Surg Traumatol..

[24] Andia I, Martin JI, Maffulli N (2018). Platelet-rich plasma and mesenchymal stem cells: exciting, but … are we there yet?. Sports Med Arthrosc Rev..

[25] Hurley ET, Hannon CP, Pauzenberger L, Fat DL, Moran CJ, Mullett H (2019). Nonoperative treatment of rotator cuff disease with platelet-rich plasma: a systematic review of randomized controlled trials. Arthroscopy..

[26] Schwitzguebel AJ, Kolo FC, Tirefort J, Kourhani A, Nowak A, Gremeaux V, Saffarini M, Lädermann A (2019). Efficacy of platelet-rich plasma for the treatment of interstitial supraspinatus tears: a double-blinded, randomized controlled trial. Am J Sports Med..

[27] Alt EU, Schmitz C, Bai X, Trafny T, Spiri S (2020). Fundamentals of Stem cells: why and how patients' own adult stem cells are the next generation of medicine. Bioethics and research on adult stem cells.

[28] Winnier GE, Valenzuela N, Peters-Hall J, Kellner J, Alt C, Alt EU (2019). Isolation of adipose tissue derived regenerative cells from human subcutaneous tissue with or without the use of an enzymatic reagent. PLoS One..

[29] Solakoglu Ö, Götz W, Kiessling MC, Alt C, Schmitz C, Alt EU (2019). Improved guided bone regeneration by combined application of unmodified, fresh autologous adipose derived regenerative cells and plasma rich in growth factors: A first-in-human case report and literature review. World J Stem Cells..

[30] Haenel A, Ghosn M, Karimi T, Vykoukal J, Shah D, Valderrabano M, Schulz DG, Raizner A, Schmitz C, Alt EU (2019). Unmodified autologous stem cells at point of care for chronic myocardial infarction. World J Stem Cells..

[31] Boutron I, Altman DG, Moher D, Schulz KF, Ravaud P, CONSORT NPT Group (2017). CONSORT Statement for randomized trials of nonpharmacologic treatments: a 2017 Update and a CONSORT extension for nonpharmacologic trial abstracts. Ann Intern Med..

[32] Chan AW, Tetzlaff JM, Altman DG, Laupacis A, Gøtzsche PC, Krleža-Jerić K, Hróbjartsson A, Mann H, Dickersin K, Berlin JA, Doré CJ, Parulekar WR, Summerskill WS, Groves T, Schulz KF, Sox HC, Rockhold FW, Rennie D, Moher D (2013). SPIRIT 2013 statement: defining standard protocol items for clinical trials. Ann Intern Med..

[33] Ben Kibler W, Sciascia AD, Hester P, Dome D, Jacobs C (2009). Clinical utility of traditional and new tests in the diagnosis of biceps tendon injuries and superior labrum anterior and posterior lesions in the shoulder. Am J Sports Med..

[34] Neer CS 2nd. Impingement lesions. Clin Orthop Relat Res. 1983;(173):70–7. 6825348

[35] European Medicine Agency. Guideline on missing data in confirmatory clinical trials, 2010 [cited 10 February 2020]. Available from: www.ema.europa.eu/docs/en_GB/document_library/Scientific_guideline/2010/09/WC500096793.pdf.

[36] Coleman WP, Hendry SL (2006). Principles of liposuction. Semin Cutan Med Surg..

[37] Klein JA (1993). Tumescent technique for local anesthesia improves safety in large-volume liposuction. Plast Reconstr Surg..

[38] Ware J, Donald Sherbourne C (1992). The MOS 36-item short-form health survey (SF-36). I. Conceptual framework and item selection. Med Care.

[39] Hays RD, Sherbourne CD, Mazel RM (1993). The RAND 36-Item Health Survey 1.0. Health Econ.

[40] Angst F, Schwyzer HK, Aeschlimann A, Simmen BR, Goldhahn J. Measures of adult shoulder function: Disabilities of the Arm, Shoulder, and Hand Questionnaire (DASH) and its short version (QuickDASH), Shoulder Pain and Disability Index (SPADI), American Shoulder and Elbow Surgeons (ASES) Society standardized shoulder assessment form, Constant (Murley) Score (CS), Simple Shoulder Test (SST), Oxford Shoulder Score (OSS), Shoulder Disability Questionnaire (SDQ), and Western Ontario Shoulder Instability Index (WOSI). Arthritis Care Res. 2011;63 Suppl 11:S174-S188. doi: 10.1002/acr.20630. 10.1002/acr.20630 22588743

[41] Wylie JD, Beckmann JT, Granger E, Tashjian RZ (2014). Functional outcomes assessment in shoulder surgery. World J Orthop..

[42] Lins L, Carvalho FM (2016). SF-36 total score as a single measure of health-related quality of life: Scoping review. SAGE Open Med..

[43] Bronstein IN, Semendjajew KA. Handbook of Mathematics (3^rd^ ed). Harri Deutsch, Frankfurt/Main, 1985.

[44] Hurd J. Autologous adult adipose-derived regenerative cell injection into chronic partial-thickness rotator cuff tears. ClinicalTrials.gov Identifier: NCT03752827 [cited 09 December 2019]. Available from: https://www.clinicaltrials.gov/ct2/show/NCT03752827.

[45] https://www.meddra.org/ [cited 10 February 2019].

[46] Aronowitz JA, Lockhart RA, Hakakian CS, Birnbaum ZE (2016). Adipose stromal vascular fraction isolation: A head-to-head comparison of 4 cell separation systems #2. Ann Plast Surg..

[47] Alt E, Milz S, Frank HG, Rothoerl R, Hoppert M, Alt C, Winnier G, Schmitz C. Rotator cuff tear treated with adipose derived regenerative cells. Submitted for publication.

[48] Bai X, Yan Y, Coleman M, Wu G, Rabinovich B, Seidensticker M, Alt E (2011). Tracking long-term survival of intramyocardially delivered human adipose tissue-derived stem cells using bioluminescence imaging. Mol Imaging Biol..

[49] Sadat S, Gehmert S, Song YH, Yen Y, Bai X, Gaiser S, Klein H, Alt E (2007). The cardioprotective effect of mesenchymal stem cells is mediated by IGF-I and VEGF. Biochem Biophys Res Commun..

[50] González MA, Gonzalez-Rey E, Rico L, Büscher D, Delgado M (2009). Adipose-derived mesenchymal stem cells alleviate experimental colitis by inhibiting inflammatory and autoimmune responses. Gastroenterology..

[51] Leto Barone AA, Khalifian S, Lee WP, Brandacher G (2013). Immunomodulatory effects of adipose-derived stem cells: fact or fiction?. Biomed Res Int..

[52] Ellera Gomes JL, da Silva RC, Silla LMR, Abreu MR, Pellanda R (2012). Conventional rotator cuff repair complemented by the aid of mononuclear autologous stem cells. Knee Surg Sports Traumatol Arthrosc..

[53] Hernigou P, Flouzat Lachaniette CH, Delambre J, Zilber S, Duffiet P, Chevallier N, Rouard H (2014). Biologic augmentation of rotator cuff repair with mesenchymal stem cells during arthroscopy improves healing and prevents further tears: a case-controlled study. Int Orthop..

[54] Havlas V, Kotaška J, Koníček P, Trč T, Konrádová Š, Kočí Z, Syková E (2015). Pouziti kultivovanych lidskych autolognich kmenovych bunek kostni drene pri rekonstrukci ruptury rotatorove manzety - studie bezpecnosti metody, predbezne vysledky [Use of cultured human autologous bone marrow stem cells in repair of a rotator cuff tear: preliminary results of a safety study] [Article in Czech]. Acta Chir Orthop Traumatol Cech..

[55] Kim YS, Sung CH, Chung SH, Kwak SJ, Koh YG (2017). Does an injection of adipose-derived mesenchymal stem cells loaded in fibrin glue influence rotator cuff repair outcomes? a clinical and magnetic resonance imaging study. Am J Sports Med..

[56] Jo CH, Chai JW, Jeong EC, Oh S, Kim PS, Yoon JY, Yoon KS (2018). Intratendinous injection of autologous adipose tissue-derived mesenchymal stem cells for the treatment of rotator cuff disease: a first-in-human trial. Stem Cells..

[57] Cossu G, Birchall M, Brown T, De Coppi P, Culme-Seymour E, Gibbon S, Hitchcock J, Mason C, Montgomery J, Morris S, Muntoni F, Napier D, Owji N, Prasad A, Round J, Saprai P, Stilgoe J, Thrasher A, Wilson J (2018). Lancet Commission: Stem cells and regenerative medicine. Lancet..

[58] Jo CH, Shin JS, Park IW, Kim H, Lee SY (2013). Multiple channeling improves the structural integrity of rotator cuff repair. Am J Sports Med..

[59] Taniguchi N, Suenaga N, Oizumi N, Miyoshi N, Yamaguchi H, Inoue K, Chosa E (2015). Bone marrow stimulation at the footprint of arthroscopic surface-holding repair advances cuff repair integrity. J Shoulder Elbow Surg..

[60] Osti L, Del Buono A, Maffulli N (2011). Microfractures at the rotator cuff footprint: a randomised controlled study. Int Orthop.

[61] Cai YZ, Zhang C, Lin XJ (2015). Efficacy of platelet-rich plasma in arthroscopic repair of full-thickness rotator cuff tears: a meta-analysis. J Shoulder Elbow Surg..

[62] Warth RJ, Dornan GJ, James EW, Horan MP, Millett PJ (2015). Clinical and structural outcomes after arthroscopic repair of full-thickness rotator cuff tears with and without platelet-rich product supplementation: a meta-analysis and meta-regression. Arthroscopy..

[63] Fu CJ, Sun JB, Bi ZG, Wang XM, Yang CL (2017). Evaluation of platelet-rich plasma and fibrin matrix to assist in healing and repair of rotator cuff injuries: a systematic review and meta-analysis. Clin Rehabil..

[64] Miranda I, Sánchez-Alepuz E, Lucas FJ, Carratalá V, González-Jofre CA (2017). Use of platelet-rich plasma in the treatment of rotator cuff pathology. What has been scientifically proven?. Rev Esp Cir Ortop Traumatol..

[65] Hurley ET, Lim Fat D, Moran CJ, Mullett H (2019). The efficacy of platelet-rich plasma and platelet-rich fibrin in arthroscopic rotator cuff repair: a meta-analysis of randomized controlled trials. Am J Sports Med..

[66] Jazrawi LM, Baron SL (2019). Editorial commentary: sprinkle some pixie dust on it-are we really any better at understanding the benefits of platelet-rich plasma for rotator cuff pathology?. Arthroscopy..

[67] Han C, Na Y, Zhu Y, Kong L, Eerdun T, Yang X, Ren Y (2019). Is platelet-rich plasma an ideal biomaterial for arthroscopic rotator cuff repair? A systematic review and meta-analysis of randomized controlled trials. J Orthop Surg Res..

[68] Murray IR, LaPrade RF, Musahl V, Geeslin AG, Zlotnicki JP, Mann BJ, Petrigliano FA. Biologic treatments for sports injuries II Think tank-current concepts, future research, and barriers to advancement, Part 2: rotator cuff. Orthop J Sports Med. 201631;4(3):2325967116636586. 10.1177/2325967116636586. 10.1177/2325967116636586 PMC4820026 27099865

[69] Coory JA, Parr AF, Wilkinson MP, Gupta A (2019). Efficacy of suprascapular nerve block compared with subacromial injection: a randomized controlled trial in patients with rotator cuff tears. J Shoulder Elbow Surg..

